# Supplementary material for: Evaluation of Transition Metal Catalysts in Electrochemically Induced Aromatic Phosphonation
Source: Molecules. 2019 May 11;24(9):1823. doi: 10.3390/molecules24091823 (PMC6540189; doi:10.3390/molecules24091823)
Supplement: Supplementary file 1 [file molecules-24-01823-s001.pdf]

Supplementary Information (ESI)

Evaluation of Transition Metal catalysts in Electrochemically Induced Aromatic Phosphonation

Sofia Strekalova, Mikhail Khrizanforov and Yulia Budnikova *

Arbuzov Institute of Organic and Physical Chemistry, FRC Kazan Scientific Center of RAS, Kazan, Russian Federation

***Correspondence:** yulia@iopc.ru

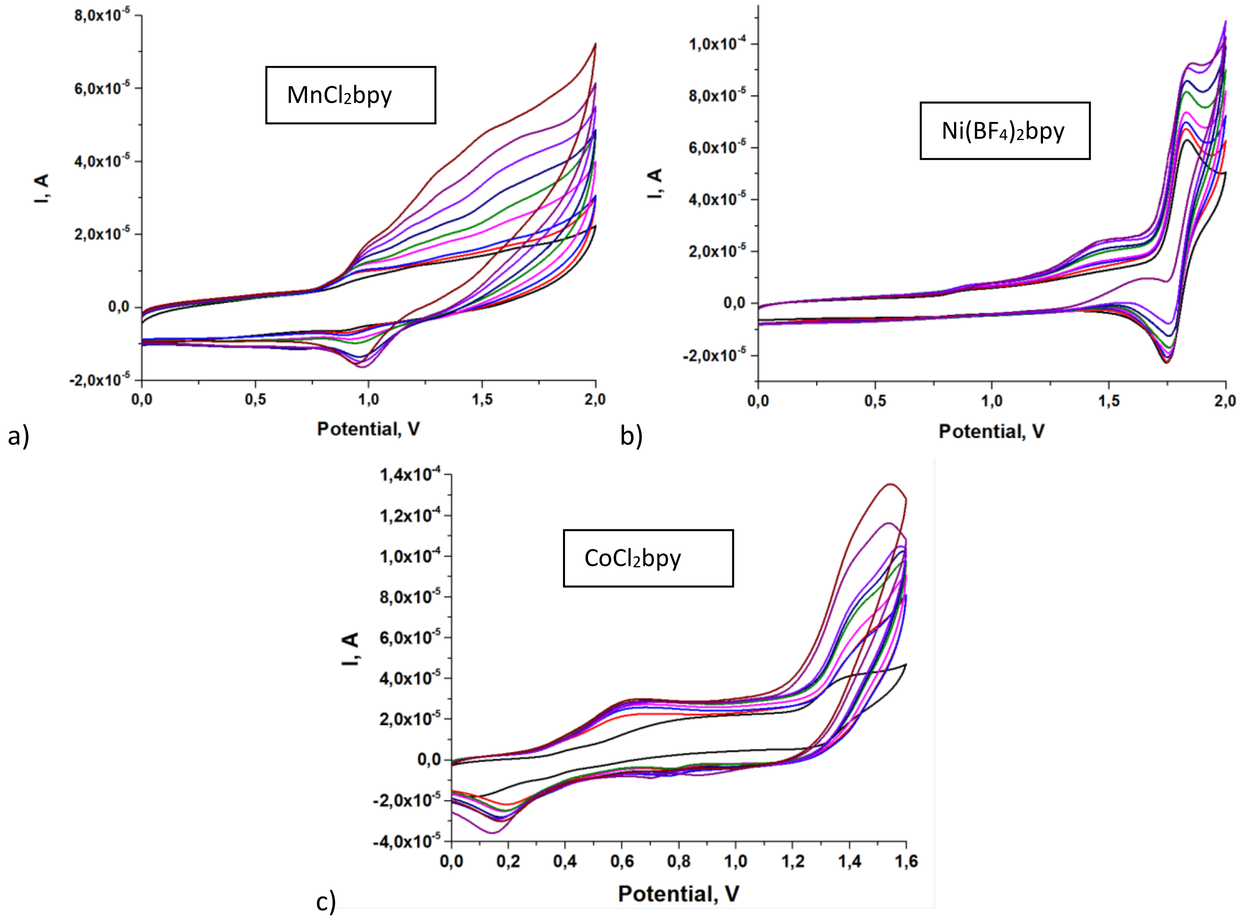

Figure S1. CVs of complexes ($5\cdot10^{-3}$ M;forMnCl$_2$bpy $1.7\cdot10^{-3}$ M) in the presence of increasing amount of DEP: **a)** MnCl$_2$bpy + HP(O)(OEt)$_2$ (1:0, 1:1, 1:6, 1:12, 1:24, 1:48, 1:96, 1:144, 1:168); **b)** Ni(BF$_4$)$_2$bpy + HP(O)(OEt)$_2$ (1:0, 1:1, 1:6, 1:12, 1:24, 1:36, 1:72, 1:144); **c)** CoCl$_2$bpy+ HP(O)(OEt)$_2$ (1:0, 1:1, 1:6, 1:12, 1:24, 1:36, 1:72, 1:108, 1:144). Conditions: CH_3CN, 0.1 V/s, 0.1 M Et_4NBF_4, Ref. electrode – Ag/AgCl, WE – GC.

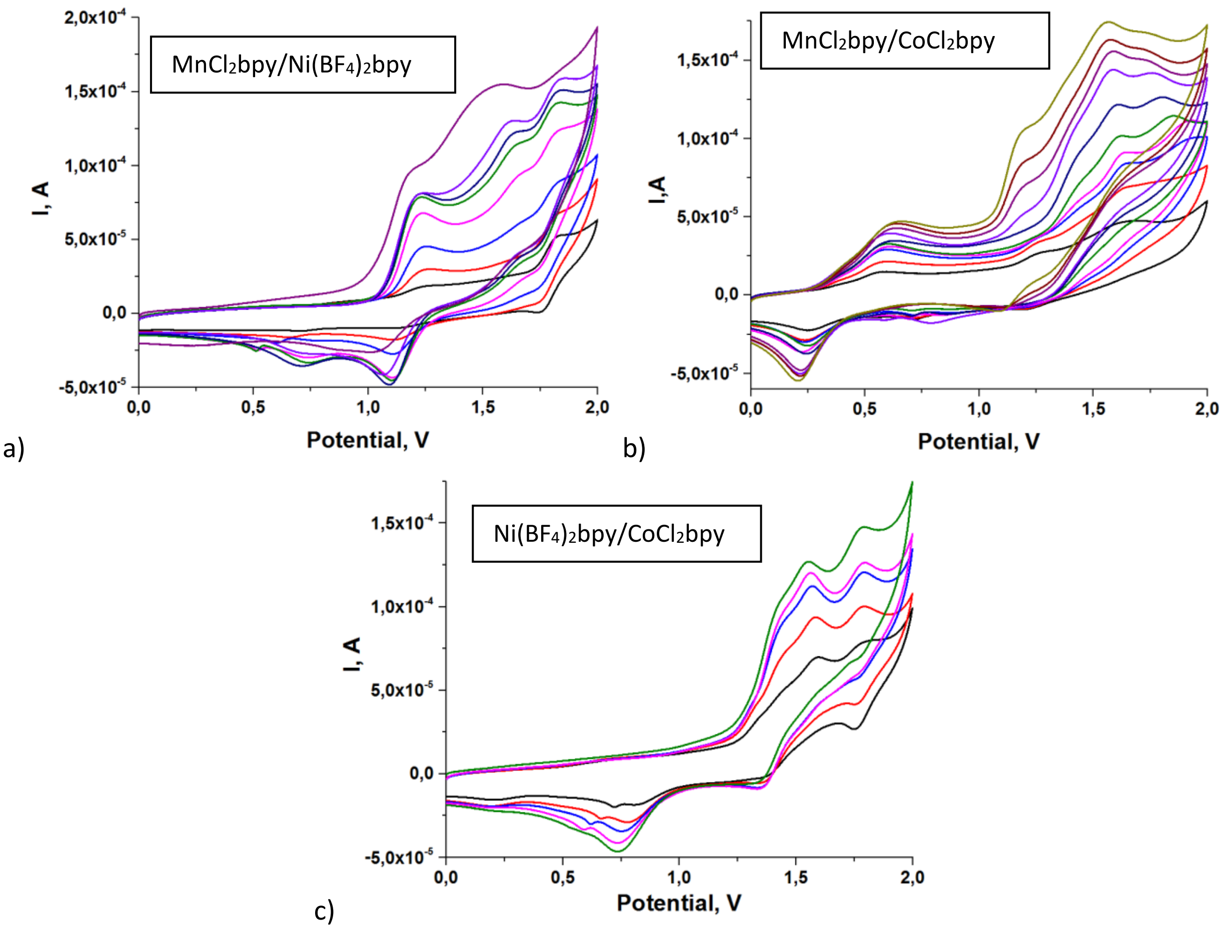

Figure S2. CVs of a mixture of complexes ($5\cdot10^{-3}$ M) in the presence of increasing amount of DEP: **a)** $MnCl_2bpy/Ni(BF_4)_2bpy$ (1:0, 1:1, 1:6, 1:12, 1:24, 1:36, 1:72, 1:108, 1:144, 1:180); **b)** $MnCl_2bpy/CoCl_2bpy$ (1:0, 1:1, 1:6, 1:12, 1:24, 1:36, 1:72, 1:108, 1:144, 1:180); **c)** $CoCl_2bpy/Ni(BF_4)_2bpy$ (1:0, 1:1, 1:6, 1:12, 1:24). Conditions: CH_3CN, 0.1 V/s, 0.1 M Et_4NBF_4, Ref. electrode – Ag/AgCl, WE – GC.

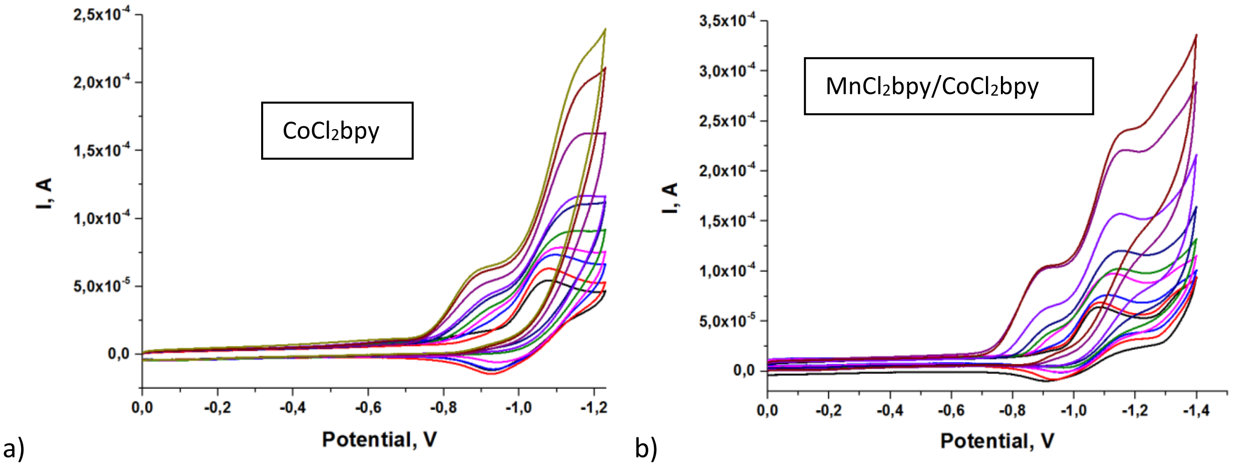

Figure S3. CVs of complexes ($5\cdot10^{-3}$ M) in the presence of increasing amount of DEP: **a)** $CoCl_2bpy$ + $HP(O)(OEt)_2$ (1:0, 1:1, 1:3, 1:6, 1:12, 1:24, 1:36, 1:72, 1:144); **b)** $MnCl_2bpy/CoCl_2bpy$ + $HP(O)(OEt)_2$ (1:0, 1:1, 1:6, 1:12, 1:24, 1:36, 1:72, 1:108, 1:144, 1:180). Conditions: CH_3CN, 0.1 V/s, 0.1 M Et_4NBF_4, Ref. electrode – Ag/AgCl, WE – GC.

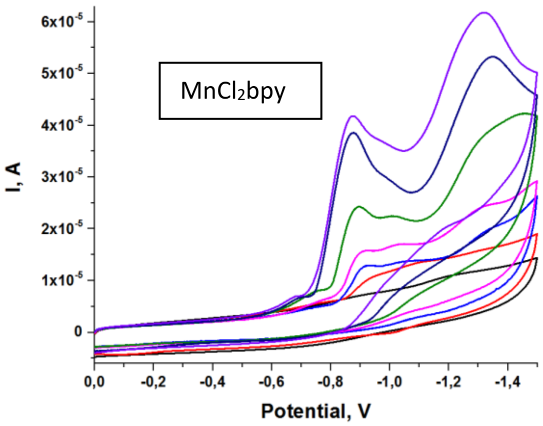

Figure S4.CVA of $MnCl_2bpy$ (1.7·10^{-3} M) in the presence of increasing amount of DEP (1:0, 1:1, 1:3, 1:6, 1:12, 1:24, 1:36, 1:72, 1:144). Conditions: CH_3CN, 0.1 V/s, 0.1 M Et_4NBF_4, Ref. electrode – Ag/AgCl, WE – CC.

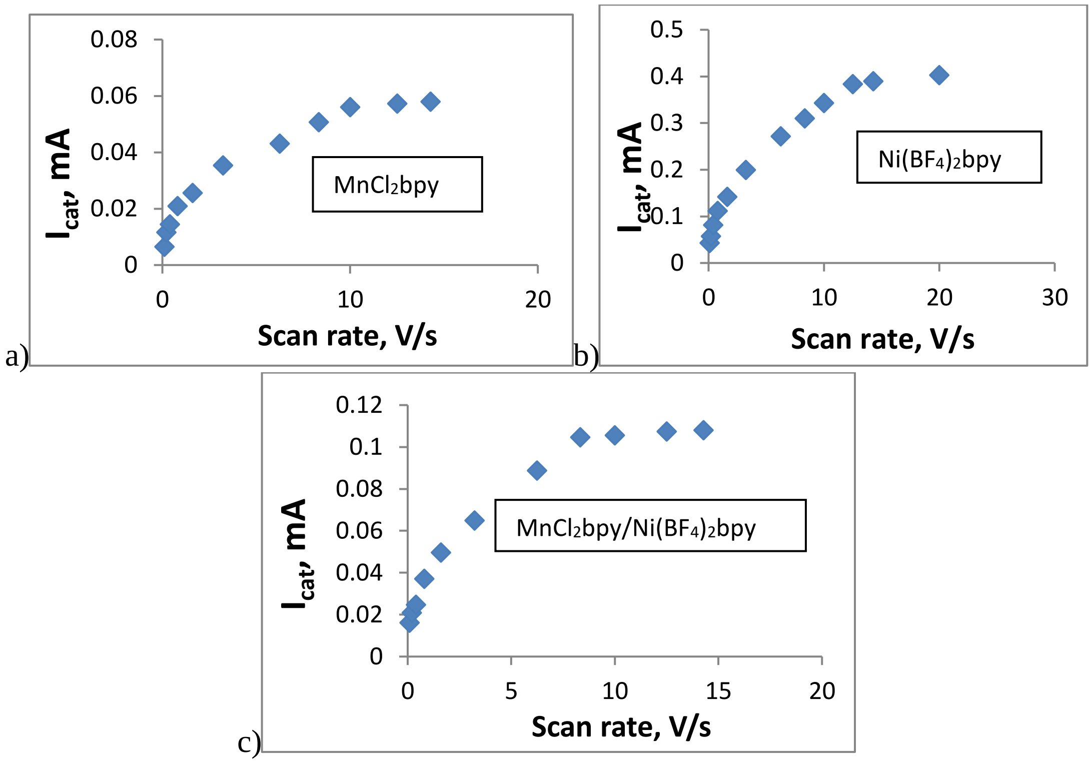

Figure S5. Dependence i_{cat} vs the scanning rate υ for the oxidation wave (in the presence of excess amount of HP(O)(OEt)2), corresponding to: **a)** Mn(II/III) [Mn(II)bpy]=1.7 M); **b)** Ni(II/III) [Ni(II)bpy]= 5·10^{-3} M); **c)** Mn(II/III)/Ni(II) [Mn(II)bpy]=[Ni(II)bpy] = 5·10^{-3} M.
